# Effects of endurance exercise on physiologic complexity of the hemodynamics in prefrontal cortex

**DOI:** 10.1117/1.NPh.11.1.015009

**Published:** 2024-03-21

**Authors:** Yinglu Hong, Dapeng Bao, Brad Manor, Yuncong Zhou, Junhong Zhou

**Affiliations:** a Beijing Sport University, School of Sport Medicine and Physical Therapy, Beijing, China; b Beijing Sport University, China Institute of Sport and Health Science, Beijing, China; c Hebrew Senior Life Hinda and Arthur Marcus Institute for Aging Research, Harvard Medical School, Boston, Massachusetts, United States; d Beijing Sport University, School of Education, Beijing, China

**Keywords:** endurance exercise, functional near-infrared spectroscopy, hemodynamics of prefrontal cortex, intaking hydrogen gas, multiscale entropy

## Abstract

**Significance:**

Prefrontal cortex (PFC) hemodynamics are regulated by numerous underlying neurophysiological components over multiple temporal scales. The pattern of output signals, such as functional near-infrared spectroscopy fluctuations (i.e., fNIRS), is thus complex. We demonstrate first-of-its-kind evidence that this fNIRS complexity is a marker that captures the influence of endurance capacity and the effects of hydrogen gas ($H_{2}$) on PFC regulation.

**Aim:**

We aim to explore the effects of different physical loads of exercise as well as the intaking of hydrogen gas on the fNIRS complexity of the PFC.

**Approach:**

Twenty-four healthy young men completed endurance cycling exercise from 0 (i.e., baseline) to 100% of their physical loads after intaking 20 min of either $H_{2}$ or placebo gas (i.e., control) on each of two separate visits. The fNIRS measuring the PFC hemodynamics and heart rate (HR) was continuously recorded throughout the exercise. The fNIRS complexity was quantified using multiscale entropy.

**Results:**

The fNIRS complexity was significantly greater in the conditions from 25% to 100% of the physical load (p<0.0005) compared with the baseline and after intaking $H_{2}$ before exercise; this increase of fNIRS complexity was significantly greater compared with the control (p=0.001∼0.01). At the baseline, participants with a greater fNIRS complexity had a lower HR (β=−0.35∼−0.33, p=0.008∼0.02). Those with a greater increase of complexity had a lower increase of the HR (β=−0.30∼−0.28, p=0.001∼0.002) during exercise.

**Conclusions:**

These observations suggest that fNIRS complexity would be a marker that captures the adaptive capacity of PFC to endurance exercise and to the effects of interventions on PFC hemodynamics.

## 1 Introduction

Endurance exercise often induces fatigue,^1^ leading to diminished performance in athletes^2^ and increased injury risk^3^ in both athletic and non-athletic populations.^4^ In addition to peripheral neurophysiological procedures (e.g., muscle contractility and excitatory contraction coupling^5^), the maintenance of performing endurance exercise depends upon the capacity of supraspinal components^6^^,^^7^ to send appropriate neural control commands to actively modulate the motor units.^8^^,^^9^ Among them, the prefrontal cortex (PFC) of the brain is the region where the information pertaining to fatigue is processed and the command to terminate exercise (i.e., inhibitory control) is initiated^10^^–^^12^ when the physical load of exercise alters the homeostasis of the individual.^13^

Studies have demonstrated that the changes in PFC activation, which can be assessed by measuring the changes in oxygenation hemodynamics within this region using functional near-infrared spectroscopy (fNIRS) technique, are linked to the performance of endurance exercise and the degree of fatigue. Specifically, the increase of PFC activation, as assessed by the increased concentration level of the oxygenated hemoglobin (i.e., ${\mathrm{HbO}}_{2}$ of fNIRS), is associated with an increase of the physical load, and inversely, the decline of oxygenated hemoglobin concentration is associated with the presence of fatigue, indicating the failure to maintain exercise.^14^^,^^15^ For example, it was observed that the capacity of elite Kenyan runners to maintain the PFC activation during self-paced five-kilometer running contributes significantly to their success in this task.^16^ In one of our previous studies,^17^ we observed that, during the performance of endurance exercise, the changes of PFC activation induced by an increased physical load were closely associated with that of the fatigue level, as quantified using the heart rate (HR).^18^^–^^20^ These kinds of evidence suggest that the regulation of the hemodynamics of PFC is critical to maintaining the performance of endurance exercise under the influences of fatigue.

Traditionally, the characterization of the hemodynamic regulation of PFC via fNIRS relies primarily on the mean or variation of the concentration level in oxygenated and deoxygenated hemoglobin (i.e., HHb of fNIRS).^21^ These measurements are based upon single scale, which, although important, do not fully characterize the hemodynamic regulation of PFC over multiple temporospatial scales. The regulation of PFC activation, indeed, depends upon numerous underlying control elements interacting across multiple scales of time and space, including the vascular tone of brain tissue at the “micro” scale,^22^^,^^23^ the cellular metabolisms at the “meso” scale, and the circadian rhythms at the “macro” scale. The dynamics of the output signals of this regulation procedure (e.g., the continuous fNIRS time series) are thus “complex,” even during the resting condition and contain rich, non-random, and meaningful information that reflects the interaction of underlying bio-physiological control elements related to the activation of PFC acting over multiple temporospatial scales.^24^^,^^25^

Recent studies have utilized techniques derived from chaos theory, such as multiscale entropy (MSE), to quantify the complexity of fNIRS signals of PFC and link it to multiple health-related conditions.^26^^,^^27^ For example, Perpetuini et al.^27^ observed that the complexity of the fNIRS signal recorded during the free and cued selective reminding test was higher for patients with early Alzheimer’s disease compared with healthy counterparts. These observations indicate that the fNIRS complexity may be a promising marker for characterizing the regulation of PFC activation that pertains to health-related conditions that the traditional measures cannot capture. However, the effects of different physical loads of endurance exercise on the fNIRS complexity of PFC, as well as the relationship between exercise-induced changes in fNIRS complexity and that of the underlying physiologic characteristics pertaining to fatigue (e.g., HR), have not been explicitly explored. Additionally, it has been shown that intaking hydrogen ($H_{2}$), an antioxidant, holds great promise for helping to alleviate exercise-induced fatigue.^28^^,^^29^ We previously observed that such effects of $H_{2}$ on fatigue are associated with its effects on the maintenance of PFC activation during the endurance exercise. Still, the effects of $H_{2}$ on the multiscale regulation of PFC hemodynamics, which can be assessed using fNIRS complexity, are unclear.

Here we aim to characterize the fNIRS complexity of PFC hemodynamics during the performance of endurance exercise based upon the data from a previous study^17^ that consists of a group of healthy younger adults. The fNIRS complexity was quantified in each condition of 0% (i.e., warm-up), 25%, 50%, 75%, and 100% of the physical loads when performing the endurance exercise with and without intaking $H_{2}$ before exercise. Specifically, we hypothesized that (1) compared with a 0% physical load, the fNIRS complexity of PFC would decrease along with the increase of the physical load; (2) compared with the control, the intake of $H_{2}$ gas may induce greater improvement in fNIRS complexity; and (3) those with a greater baseline fNIRS complexity would have lower fatigue during exercise.

## 2 Materials and Methods

### 2.1 Participants

Twenty-four young men were recruited from Beijing Sport University. The sample size was determined using PASS version 15.0 (NCSS, LLC, Kaysville, Utah). Specifically, using repeated measures, within-between interaction ANOVA models at the expected alpha (α) level of 0.05 and the desired power (1-β) of 0.9 indicated a minimum sample size of 17 participants to enable the detection of a statistically-significant effects. Considering a 30% drop-off rate, 24 participants were needed. The inclusion criteria were as follows: (1) age between 18 and 35 years and (2) the ability to complete the incremental exercise test and the cycling time to exhaustion prior to the formal trial. The exclusion criteria were as follows: (1) a self-reported acute illness, injury, or unstable medical condition or hospitalization within the past 3 months; (2) report of any conditions in the musculoskeletal system (e.g., pain or orthopedic problems) that may affect the exercise performance; and (3) the use of antipsychotics, anti-seizure, or other neuroleptic medication. Before the study visits, participants were instructed to refrain from exercise that might cause fatigue for 48 h and alcohol and caffeine intake for 24 h. All participants were informed of the relevant benefits and possible risks involved in participating in this study and provided signed informed consent to participate in this study. The consent form included information contained in the Helsinki Declaration as well as the purpose of the study and details of the study’s protocols. This study was reviewed and approved by the Institutional Review Board of Beijing Sport University (number: 2021163H).

### 2.2 Study Protocol

The specific study protocol was demonstrated in our previous paper.^17^ Each participant completed four study visits. During the first visit, an exercise was performed until exhaustion on an electromagnetically braked cycle ergometer (Excalibur Sport, Lode, Groningen, Netherlands) to measure the peak power output of each participant. After an interval of at least 48 h, on the second visit, participants performed a time-to-exhaustion cycling test to measure their maximum riding time (MRT). The test consisted of a 3 min warm-up at 40% of peak power output followed by a rectangular workload corresponding to 80% of peak power output that was achieved in the first visit. Then on the following two visits, participants completed the cycling test and functional assessments after intaking $H_{2}$ gas or placebo gas (as designed in a randomized order).^27^ These two visits were separated by one week to avoid potential after effects of the interventions from the prior visit.^17^ Specifically, on each of these two visits, participants first inhaled $H_{2}$ gas (i.e., $H_{2}$ group) or placebo gas (i.e., control group) for 20 min.^30^ After the inhalation, participants were asked to sit quietly for 2 min to record the baseline resting-state fNIRS data. Then after warming up at a load of 40% peak power output for 3 min (i.e., 0% of the physical load), participants were instructed to ride at a workload corresponding to 80% of peak power output for their MRT, which was considered to be the protocol of maximal physical load to each participant (i.e., peak power multiplied by MRT).^31^ The HR and fNIRS time series of PFC were recorded. The whole test duration was then separated into four periods of different physical loads, i.e., from 0% to 25% (i.e., 25% of the physical load), 25% to 50% (i.e., 50% physical load), 50% to 75% (i.e., 75% physical load), and 75% to 100% (i.e., 100% physical load) of the maximum workload.

### 2.3 Assessment of fNIRS

**Fig. 1:**
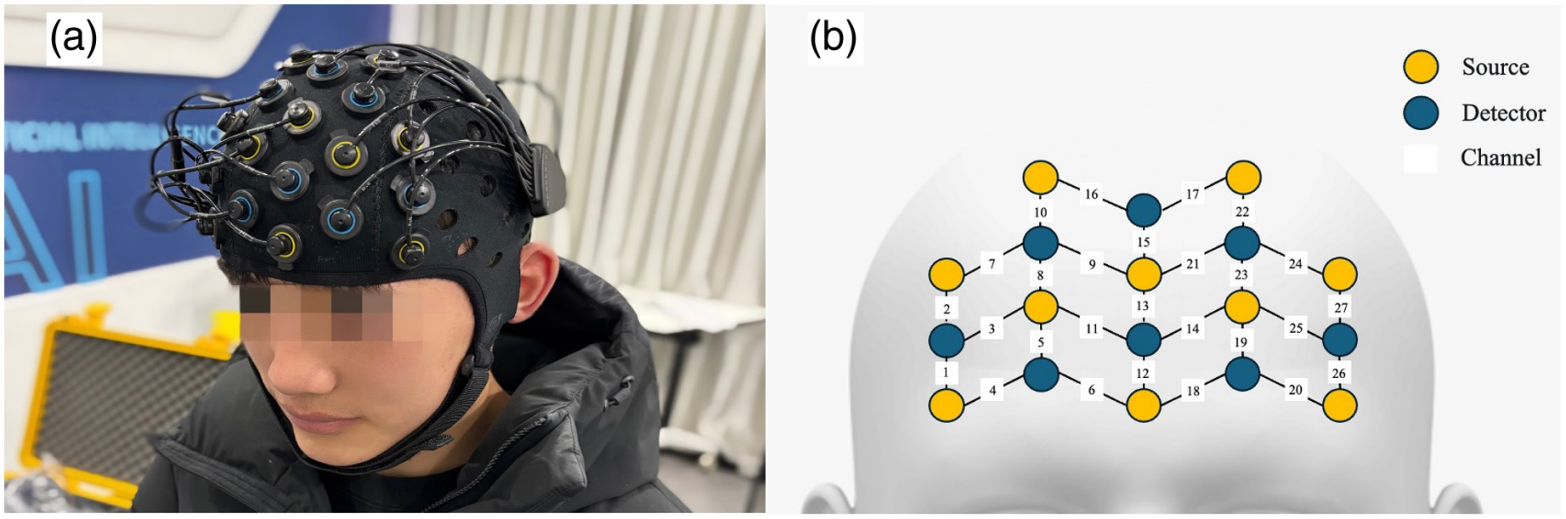
fNIRS montage. (a) Anonymized photo of a subject wearing fNIRS probes; (b) schematic diagram showing the configuration of the fNIRS probes.

The fNIRS signals of both oxygenate (${\mathrm{HbO}}_{2}$) and de-oxygenated (HHb) hemoglobin of PFC were recorded throughout the tasks using a multichannel continuous-wave fNIRS device (Oxymon, Artinis, Netherlands) consisting of 10 light sources and eight detectors mounted on a head cap. The head cap placement was centered around Cz (10/ 20 international system for electrode placement), the mid-point between the nasion to inion and left to right preauricular distances. Figure 1 shows the fNIRS montage including the placement of the fNIRS probes. The signals of the fNIRS were then obtained via 27 channels (including 12 in the right PFC and 12 in the left PFC) of the device. The sampling frequency was 10 Hz. To avoid the potential influences of the prior task condition (e.g., the physical load in the prior) on the following signals, the data of two minutes with the length of 1200 points in the middle of each physical load condition were extracted and used in the following analyses.

### 2.4 Assessment of Fatigue Using Heart Rate

We used the Firstbeat HR belt (Firstbeat Analytics, Jyvaskyla, Finland) to monitor the HR of each participant during the endurance cycling exercise. The HR belt was worn on the participant’s chest, and the receiver was positioned to the left of the midline. The elastic band was adjusted, so the position of the receiver did not change during the ride. During exercise, cardiovascular restriction is an important factor in fatigue.^32^ Studies have shown that the HR is closely associated with both exercise intensity^33^^,^^34^ and fatigue.^18^^–^^20^ Thus, we thus use the average HR within each condition of the physical load to assess fatigue.

### 2.5 Data Processing and Analysis

The recorded time series of ${\mathrm{HbO}}_{2}$ and HHb fluctuations were first pre-processed following well-established protocol^17^ as follows: (1) the relative coefficient of variation (CV in %) of the raw data in each channel was first calculated to estimate the data quality, and the data with CVs above 15% were rejected;^35^ (2) the large drifts of the signal were then removed by applying a first-order detrend;^36^ (3) the time derivative distribution repair (TDDR) algorithm was employed for the motion correction;^37^ and (4) a third-order low-pass filter with the cutoff threshold of 0.2 Hz was used to remove the noise fluctuations that were not related to the actual PFC hemodynamics.^38^ After these pre-processing steps, the average level of the first 2-min resting-state signals before exercise was obtained. The relative concentration changes of oxyhemoglobin ($\Delta {\mathrm{HbO}}_{2}$) and deoxyhemoglobin (ΔHHb) from the averaged baseline value to the performance of endurance exercise were then obtained and used in the following analyses.

#### 2.5.1 Multiscale entropy

The complexity of the pre-processed time series of $\Delta {\text{H}\mathrm{bO}}_{2}$ and ΔHHb within each physical load condition was then quantified using MSE. Specifically, the pre-processed time series of each channel was first “coarse-grained” from scale 1 to 5 by dividing the original time series into non-overlapping windows of length equaling a scale factor from 1 to 5 sampling points.^39^ For example, the series at scale 1 was the original time series consisting of 1200 points; at scale 5, the coarse-grained series was constructed by averaging every five non-overlapping points, thereby creating a new series of 240 points (i.e., 1200 points/5). Next, the sample entropy of the coarse-grained series at each scale was caculated, as defined by the negative of the natural logarithm of the conditional probability that a series, having repeated itself for m consecutive data points (m is the length of the pre-defined pattern, and the repeatability is determined by the number of patterns in this series with a difference from the pre-defined pattern smaller than the standard deviation of this series multiplied by a tolerance parameter, r), will also repeat itself for m+1 points within the same tolerance without self-matches.^39^^–^^41^ Following the same procedure of MSE calculation in previous studies,^39^^,^^41^^,^^42^ we computed the sample entropy of each coarse-grained series by choosing the parameter of tolerance r=0.15 and the number of matching points m=2. Notably, to obtain a reliable estimation of entropy, it is recommended that the number of data points in the coarse-grained time-series at the largest scale should be at least $10^{m}$ to $17^{m}$ (here m=2, so n=100∼289).^40^ On the maximum scale here (i.e., scale 5), the number of points was 240, greater than the recommended number, which suggests that the estimation of the entropy was reliable.

The averaged entropy from scale 1 to 5 was then obtained for the time series of each channel. The primary outcome was the complexity of $\Delta {\mathrm{hbO}}_{2}$ and ΔHHb signals of the whole PFC by averaging the entropy across all 27 channels. Second, we also obtained the fNIRS complexity within the left and right hemispheric PFC to explore the potential hemispheric differences.

### 2.6 Statistical Analysis

Statistical analyses were performed using JMP 16 (SAS Institute, Cary NC). The significance level of the analyses was set at p<0.05. The normality of the data was examined using the Shapiro–Wilk test, and the homogeneity of variance was examined using Levene’s test.

To examine the effects of the physical load on the fNIRS complexity, we used one-way ANOVA models within the control group when the data were normally distributed. The model factor was the physical load (i.e., resting, warm-up, 25%, 50%, 75%, and 100%), and the dependent variable was the primary outcome, i.e., the complexity of $\Delta {\mathrm{hbO}}_{2}$ and ΔHHb of the whole PFC in separate models. Tukey’s post-hoc analysis was used to compare the factor means when a significance was observed. When data were not normally distributed, we used the Kruskal–Wallis test. Second, similar models were also used to examine the effects of the physical load on the fNIRS complexity within the left and right prefrontal regions.

Then, to examine the relationships between fNIRS complexity and the degree of exercise-induced fatigue, linear regression models were used. First, the relationships between the $\Delta {\mathrm{hbO}}_{2}$ and ΔHHb complexity and the HR at the baseline were examined in separate models. The group was included as a covariate in the models. Then, within the control group, the relationships between the percent change of $\Delta {\mathrm{HbO}}_{2}$ and ΔHHb complexity and that of HR as induced by physical loads were examined. The physical load was included as the covariate in the models. Second, similar models were used to examine the relationships between the fNIRS complexity of each hemispheric PFC and HR. The hemispheric side (i.e., left and right) was included in the model to examine if there is any significant difference in the observations between the left and right PFC.

Next, to examine the effects of the $H_{2}$ gas intake on fNIRS complexity, we first calculated the percent change of the $\Delta {\mathrm{HbO}}_{2}$ and ΔHHb complexity from 0% load to other loads (i.e., 25%, 50%, 75%, and 100%). A one-way repeated-measures ANOVA model was used when the data were normally distributed. The model factor was the group of intervention (i.e., $H_{2}$ and control), and the dependent variable was the percent change of $\Delta {\mathrm{HbO}}_{2}$ and ΔHHb complexity in separate models. The level of the physical load and its interaction with the group was also included in the model to examine the potential significant contributions of any specific physical load to the effects of $H_{2}$ on fNIRS complexity. The hemispheric side (i.e., left and right) was included in the model to examine if there is any significant difference in the observations between the left and right PFC.

When data were not normally distributed, we used the Kruskal–Wallis test. Second, similar models were also used to examine the effects of $H_{2}$ gas on the fNIRS complexity within the left and right prefrontal regions.

## 3 Results

All 24 male participants (age: 21.33±2.68 years; BMI: $22.45\pm 1.93\;\mathrm{kg}/m^{2}$; peak power output: 209.17±30.35  w; maximal riding time: 20.48±8.27  min) successfully completed this study. However, the signal quality of the fNIRS channel was poor in three participants, so the data of 21 participants were included in the analyses.

Table 1 shows the mean and standard deviation of $\Delta {\mathrm{HbO}}_{2}$ and ΔHHb complexity in the $H_{2}$ and control groups in different physical load conditions. No significant differences in the fNIRS complexity or HR were observed at a physical load between the $H_{2}$ and control groups (p>0.13).

**Table 1:** fNIRS complexity in the $H_{2}$ and control groups in different physical loads.

	$H_{2}$ group					Control group				
	0%	25%	50%	75%	100%	0%	25%	50%	75%	100%
$\Delta {\mathrm{HbO}}_{2}$										
Whole	0.98 ± 0.24	1.47 ± 0.15	1.60 ± 0.17	1.60 ± 0.19	1.63 ± 0.17	0.86 ± 0.43	1.27 ± 0.45	1.41 ± 0.51	1.40 ± 0.49	1.41 ± 0.44
Left	0.99 ± 0.27	1.45 ± 0.20	1.63 ± 0.19	1.59 ± 0.25	1.61 ± 0.21	0.85 ± 0.42	1.27 ± 0.46	1.40 ± 0.51	1.39 ± 0.50	1.41 ± 0.46
Right	0.99 ± 0.34	1.38 ± 0.38	1.55 ± 0.42	1.51 ± 0.44	1.50 ± 0.43	0.89 ± 0.43	1.27 ± 0.45	1.43 ± 0.52	1.41 ± 0.51	1.43 ± 0.44
ΔHHb										
Whole	0.94 ± 0.26	1.49 ± 0.18	1.59 ± 0.15	1.56 ± 0.23	1.55 ± 0.18	0.86 ± 0.39	1.31 ± 0.38	1.44 ± 0.44	1.49 ± 0.35	1.49 ± 0.34
Left	0.89 ± 0.24	1.47 ± 0.23	1.58 ± 0.17	1.56 ± 0.25	1.57 ± 0.20	0.82 ± 0.38	1.33 ± 0.28	1.43 ± 0.43	1.45 ± 0.46	1.51 ± 0.36
Right	0.99 ± 0.30	1.52 ± 0.16	1.62 ± 0.16	1.58 ± 0.21	1.55 ± 0.20	0.89 ± 0.40	1.30 ± 0.42	1.47 ± 0.47	1.46 ± 0.48	1.49 ± 0.34

### 3.1 Effects of Physical Loads on fNIRS Complexity

**Fig. 2:**
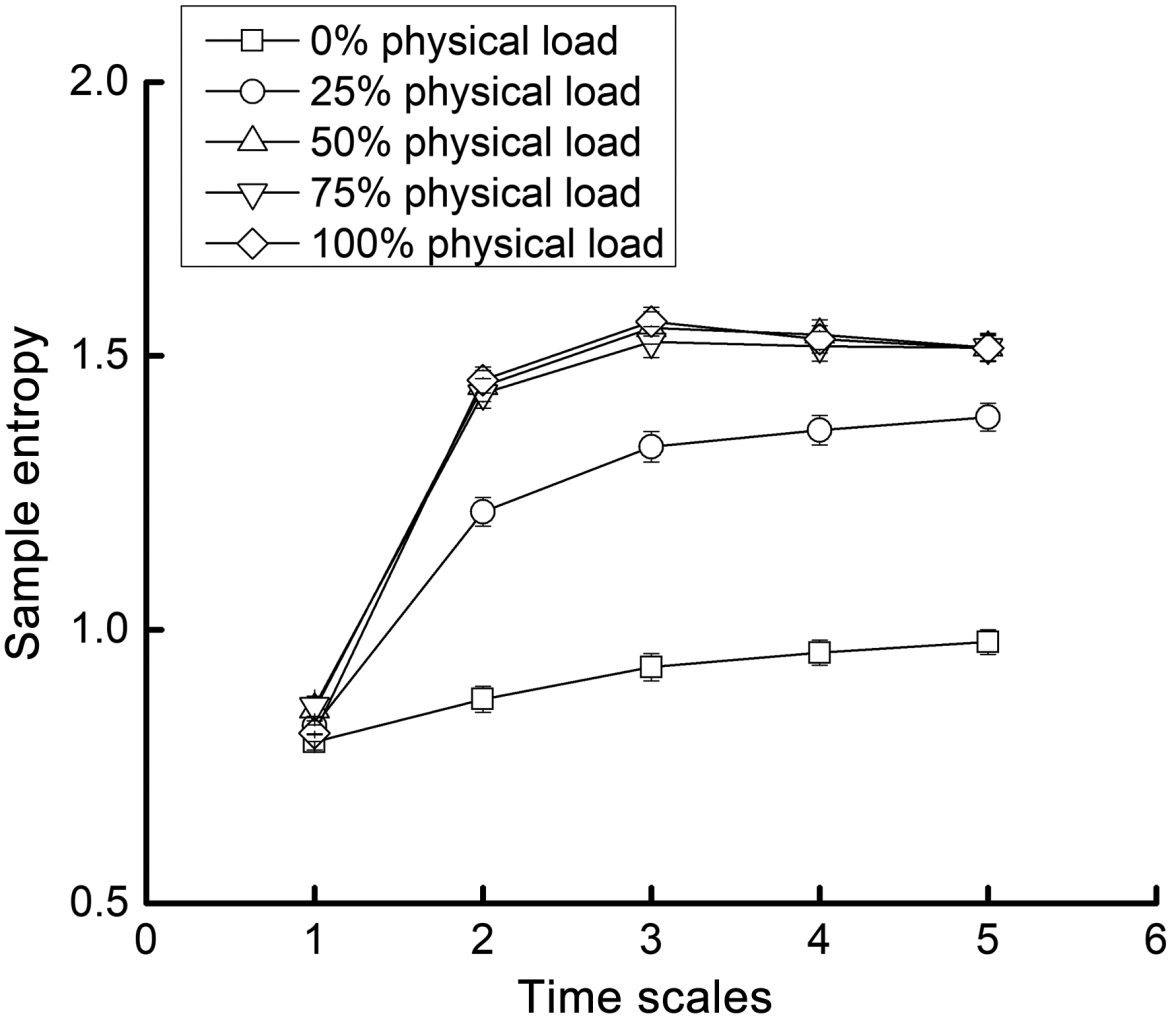
MSE curves (mean and standard error) of $\Delta {\mathrm{HbO}}_{2}$ in each condition of physical loads (i.e., 0%, 25%, 50%, 75%, 100%). Entropy at scale 1 (i.e., traditional sample entropy) was relatively similar between conditions and appeared to be different at larger scales.

Figure 2 shows the average and standard error of MSE curves of $\Delta {\mathrm{HbO}}_{2}$ in different physical load conditions of the control group. It is observed that the sample entropy at scale 1 was relatively similar between different physical loads, but starting from scale 2, they were obviously different. A greater physical load was associated with a greater MSE.

One-way ANOVA models demonstrated significant effects of the physical loads on the complexity of both $\Delta {\mathrm{HbO}}_{2}$ (p=0.0005) and ΔHHb (p<0.0001). The Tukey’s post-hoc analysis revealed that, compared with the condition of a 0% physical load, the complexity was significantly greater when participants exercised with physical loads (i.e., 25%, 50%, 75%, and 100% of the physical load), and no significant difference was observed between these four physical loads (Table 1).

Second, similar ANOVA models showed significant effects of the physical loads on the complexity of both $\Delta {\mathrm{HbO}}_{2}$ (left: p=0.0006; right: p=0.0006) and ΔHHb (left: p<0.0001; right: p<0.0001) within the left or right hemispheric PFC. Tukey’ post-hoc analyses also revealed that, compared with the condition of a 0% physical load, the complexity significantly increased when participants exercised with physical loads (Table 1).

### 3.2 Relationship between fNIRS Complexity and Fatigue

**Fig. 3:**
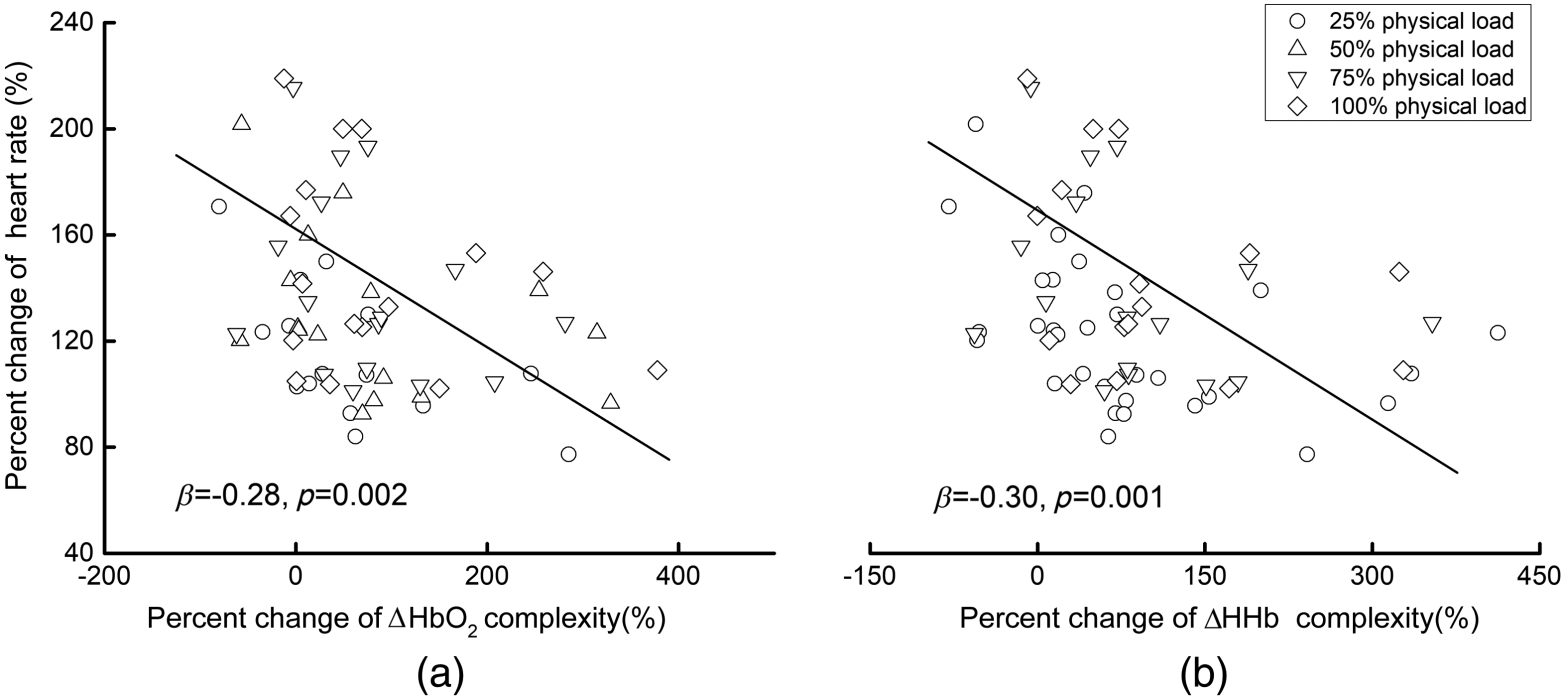
(a) The association between the percent change of $\Delta {\mathrm{HbO}}_{2}$ and (b) ΔHHb complexity and that of HR within the control group. The linear regression models showed that, within the control group, participants with a greater increase of $\Delta {\mathrm{HbO}}_{2}$ (β=−0.28, p=0.002) and/or ΔHHb (β=−0.30, p=0.001) complexity had a smaller increase of HR during the endurance exercise, indicating a lower level of fatigue. These associations were independent of the influence of the physical load condition.

The linear regression models adjusted for the group showed significant associations between the $\Delta {\mathrm{HbO}}_{2}$ (β=−0.35, p=0.008) and ΔHHb (β=−0.33, p=0.02) complexity and HR at a 0% physical load, i.e., participants with a greater fNIRS complexity had a lower HR (Fig. 3). Such associations were independent of group (p=0.12∼0.13). Second, the fNIRS complexity of both the left and right PFC was also significantly associated with the HR (β=−0.35∼−0.47, p=0.003∼0.01), and no significant effects of the group or hemispheric side on such relationships were observed (p=0.22∼0.89).

**Fig. 4:**
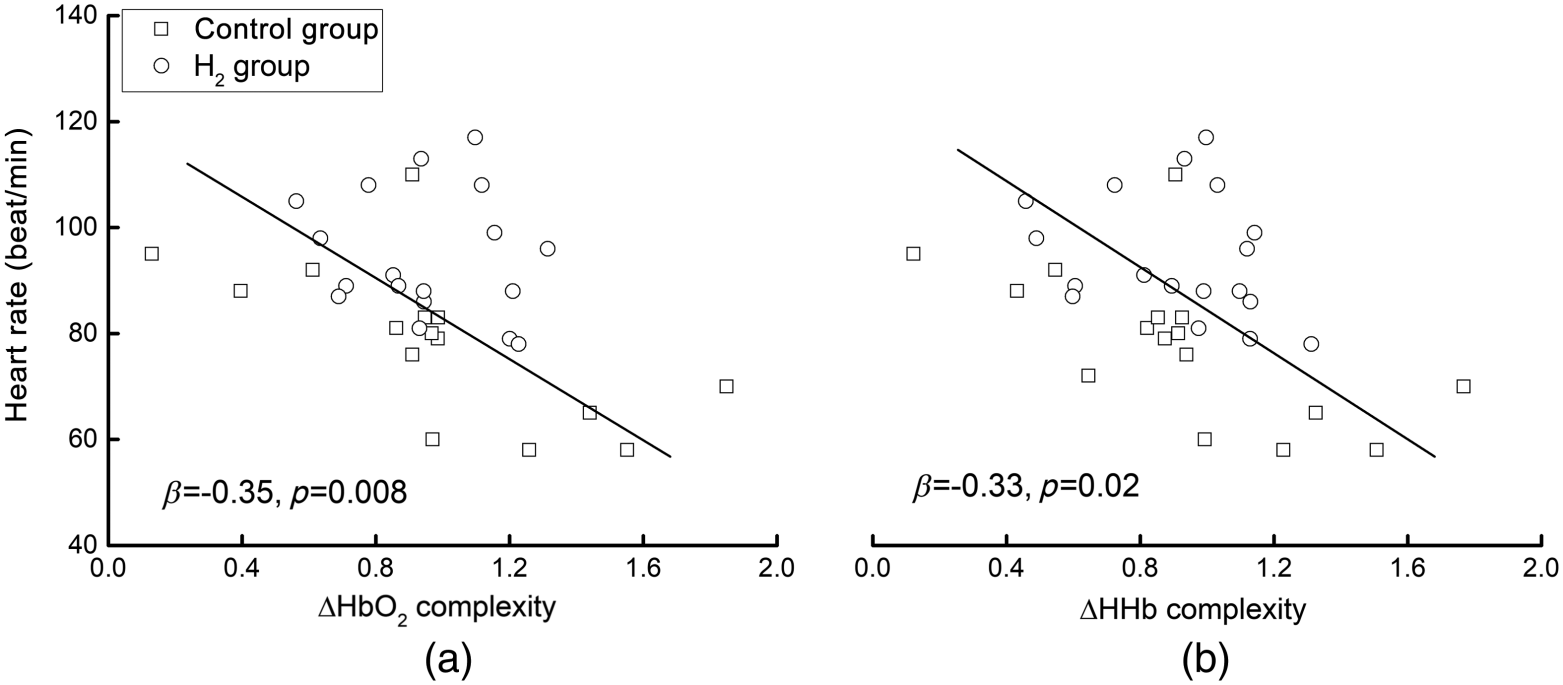
(a) The association between $\Delta {\mathrm{HbO}}_{2}$ and (b) ΔHHb complexity and HR in the condition of a 0% physical load. The linear regression models showed that, across both the $H_{2}$ and control groups, participants with greater $\Delta {\mathrm{HbO}}_{2}$ (β=−0.35, p=0.008) and/or ΔHHb (β=−0.33, p=0.02) complexity had a lower HR, respectively. Such a significant association was independent of the group.

Then the linear regression models adjusted for physical load showed that, within the control group, the percent changes of both $\Delta {\mathrm{HbO}}_{2}$ and ΔHHb complexity, as induced by the physical load, were associated with that of HR ($\Delta {\mathrm{HbO}}_{2}$: β=−0.28, p=0.002; ΔHHb: β=−0.30, p=0.001) (Fig. 4), i.e., those with a greater increase of the fNIRS complexity had a lower increase of HR during the endurance exercise (i.e., less fatigue). Such relationships were independent of the level of the physical load (p=0.19∼0.25). Second, similar relationships between the percent change of fNIRS complexity with the left and right PFC and that of HR (β=−0.35∼−0.29, p=0.001∼0.008) were also observed. Neither the level of the physical load nor the hemispheric side had significant effects on such relationships (p=0.23∼0.37).

### 3.3 Effects of the $H_{2}$ Gas Intake on fNIRS Complexity

The percent changes of $\Delta {\mathrm{HbO}}_{2}$ and ΔHHb complexity are shown in Table 2. One-way ANOVA models demonstrated significant effects of the group on the percent change of both $\Delta {\mathrm{HbO}}_{2}$ (p=0.001) and ΔHHb (p=0.01) complexity. The percent change of complexity in the $H_{2}$ group was significantly greater compared with that of the control group (Table 2). No significant effects of the physical load or its interaction with the group (p=0.12∼0.98) on fNIRS complexity were observed, indicating that there were no significant contributions from any specific physical load condition to the observations.

**Table 2:** Percent change of fNIRS complexity of different physical loads in the $H_{2}$ and control groups.

%	$H_{2}$ group				Control group			
	25%	50%	75%	100%	25%	50%	75%	100%
$\Delta {\mathrm{HbO}}_{2}$								
Whole	59.55 ± 44.93	75.09 ± 45.02	75.71 ± 46.15	78.29 ± 47.98	29.09 ± 50.50	45.65 ± 60.09	60.89 ± 0.71.55	50.91 ± 60.79
Left	58.81 ±55.09	81.06 ± 61.96	77.99 ± 65.54	78.75 ± 61.51	33.42 ± 50.95	45.78 ± 56.06	64.61 ± 71.42	55.79 ± 65.11
Right	47.91 ± 49.24	67.76 ± 55.51	63.67 ± 52.12	63.13 ± 54.16	25.36 ± 52.48	47.47 ± 69.42	58.84 ± 73.42	48.13 ± 60.35
ΔHHb								
Whole	72.45 ± 57.54	87.81 ± 58.27	82.35 ± 56.49	84.41 ± 64.96	37.03 ± 52.23	52.17 ± 59.09	65.39 ± 70.47	64.32 ± 58.22
Left	76.41 ± 57.06	93.59 ± 54.65	90.58 ± 59.82	94.23 ± 65.52	47.38 ± 56.87	56.91 ± 60.05	73.39 ± 73.59	72.94 ± 60.06
Right	67.47 ± 60.63	82.28 ± 65.25	76.09 ± 60.03	76.14 ± 68.37	29.31 ± 51.52	49.20 ± 60.48	60.94 ± 69.85	58.97 ± 58.09

Similarly, the secondary ANOVA models showed that the percent change of both $\Delta {\mathrm{HbO}}_{2}$ (p=0.0002∼0.001) and ΔHHb (p<0.0001) complexity within the left and right PFC in the $H_{2}$ group was significantly greater compared with that of the control group (Table 2), and no significant effects of the physical load and its interaction with the group (p=0.36∼0.99) were observed. Within the $H_{2}$ group, a greater percent increase of complexity was observed in the left PFC compared with the right side (Table 2), but no significant difference was observed ($\Delta {\mathrm{HbO}}_{2}$: p=0.12, ΔHHb: p=0.15).

## 4 Discussion

To our knowledge, for the first time, we characterize the multiscale dynamics of the PFC oxygenation activities in different physical loads during the performance of endurance exercise using the complexity of continuous fNIRS fluctuations in this region. The findings of this pilot study demonstrate that, in healthy younger adults with the capacity to successfully complete the endurance task, the fNIRS complexity increases when exercising, intaking $H_{2}$ gas before exercise can induce a greater improvement of the fNIRS complexity, and the fNIRS complexity is closely associated with the degree of fatigue as assessed by the HR. Taken together, these results suggest that a more complex regulation of the PFC is critical to maintaining the performance of an endurance task and that the fNIRS complexity may capture the influences of exercise load and the benefits of interventions on this important neurophysiological procedure in humans.

The results of this study reveal that, as we expected, at 0% of the physical load, participants with a greater fNIRS complexity had a lower HR. Studies have linked a lower HR, within the normal range, to better cardiovascular function pertaining to blood supply.^43^^,^^44^ This function is critical to the cerebral autoregulation and perfusion pertaining to the PFC hemodynamics. Therefore, the observation here suggests that greater fNIRS complexity of PFC captures better a cardiovascular function pertaining to the cerebral regulation. On the other hand, not as we hypothesized, healthy younger adults presented with a significantly greater fNIRS complexity of the PFC during the performance of endurance exercise. Greater complexity in the output fluctuation of a neurophysiological system or procedure is directly associated with a better capacity of that system/procedure to adapt to stressors/perturbations.^45^ Previous studies have suggested that, compared with the resting state, more neural resources, such as greater cortical activation, are devoted to maintaining the task performance.^36^^–^^48^ Our study consists of a group of healthy younger adults with an intact adaptive capacity to the “stressor” (i.e., all of them successfully completed the endurance exercise task); therefore, the exercised-related increase of fNIRS complexity of the PFC suggests that a more complex regulation of the PFC oxygenation hemodynamics pertaining to performing the endurance task is presented in this cohort. This is further supported by the evidence that a greater increase of the fNIRS complexity is associated with a lower increase of HR (i.e., less fatigue). This indicates that, even in this healthy cohort, individuals who can initiate a more complex regulation of the PFC when starting to exercise (i.e., a greater increase of fNIRS complexity) may have a greater adaptive capacity to exercise, thus presenting with a lower degree of fatigue.

Numerous studies have demonstrated that aging and/or age-related conditions often alter the multiscale regulation of the biophysiological system/procedures of humans, leading to the diminished capacity of the system to adapt to stressors or perturbations, which can be captured by a decreased physiologic complexity in the systems’ output fluctuation.^49^^–^^51^ On the other hand, evidence has also shown that such a loss of complexity is not obligatory, but it can be restored or improved via appropriate strategies. For example, Zhou et al.^52^ showed that applying sub-sensory vibratory random stimuli on foot soles can increase the complexity of the standing postural sway in older adults and such an increase of complexity is associated with the improvement of mobility. We here observe that, compared with the control, intaking the $H_{2}$ gas can induce a greater improvement in the fNIRS complexity of the PFC when performing the endurance exercise, suggesting that $H_{2}$ may be an appropriate strategy for enhancing the activation of PFC that pertains to the maintenance of endurance performance, as we demonstrated in our previous publication,^17^ by facilitating more complex regulation in the PFC hemodynamics. Thus, in future studies, exploring how the intake of $H_{2}$ influences the fNIRS complexity of the PFC in older adults or those with limited capacity of the cortical control (e.g., cognitive impairment) to appropriately allocate the neural resources to different tasks is required.

One potential underlying bio-physiological mechanism of such $H_{2}$-induced benefits may be the antioxidant effects of $H_{2}$. The exceeded reactive oxygen species (ROS) as induced by the endurance exercise often damage the neurons and mitochondrial membranes of the brain, leading to a diminished neural efficiency that is critical to the regulation of exercise performance. Evidence has been shown that $H_{2}$ can selectively scavenge ROS^53^ by successfully crossing the blood-brain barrier,^54^ helping to improve mitochondrial function and increase the efficiency of the brain, which thus benefits such a complex regulation of the PFC, as captured by the increase of the fNIRS complexity in this region. Future studies are thus warranted to explicitly measure the underlying bio-neurophysiological characteristics (e.g., mitochondrial function) related to endurance exercise, enabling the exploration of the underlying pathological mechanism through which the $H_{2}$ can help augment the multiscale complex regulations of the PFC during the endurance exercise.

We here also compared the potential hemispheric difference in the fNIRS complexity. The fNIRS complexity of both the left and right PFC is significantly increased, but a greater increase of the fNIRS complexity within the left PFC is observed compared with the right (Table 2), although the difference is not statistically significant (p=0.12∼0.15) (may be due to the lack of enough statistical power). Studies have shown that the PFC is involved in the control of motivation that allows the continuation of the motion/movement by suppressing those peripheral signals from stopping the movement (e.g., fatigue).^55^^,^^56^ Moreover, recent evidence has shown that, uniquely, the left hemispheric PFC may contribute more to such inhibitory control^57^^,^^58^ than the right PFC. For example, Rubia and colleagues observed that, in a group of healthy adults, the activation within the left, not the right PFC, as assessed by an increased blood-oxygenation-level-dependent signal of functional magnetic resonance imaging, was associated with the inhibitory control performance of the go/no-go task.^58^ Our findings may thus provide novel but preliminary support to the particular role of the left PFC in the inhibitory control. Still, future studies with large sample sizes are needed to examine and confirm this potential hemispheric difference in the function of PFC. With this knowledge, a more in-depth understanding of the regional characteristics of the supraspinal regulation of endurance exercise can be had, ultimately helping the design of training and rehabilitative strategies with a more appropriate target for endurance exercise and its related fatigue.

Some limitations should be noted in this pilot study. Due to the relatively small sample size, we did not perform the channel-based analysis of fNIRS complexity but characterized it only for the whole PFC and each hemisphere. The fNIRS probes were placed following the 10/20 template across all participants. Using the spatial registration method for each participant may provide more consistent and precise placement.^59^ Therefore, future studies with greater sample size and using the spatial registration technique are warranted to ensure more consistent and precise probe placement and help characterize the topographical map of the fNIRS complexity of the PFC and other related regions that may also contribute to the endurance performance (e.g., primary motor cortex). Only healthy younger men were included in this study; therefore, the potential influences of sex on the observation and how physical loads influences the fNIRS complexity in other cohorts (e.g., those in the recovery period of sports injury) were not explored. Additionally, only the immediate effects of one-dose $H_{2}$ on the fNIRS complexity were examined. Future studies implementing a longer term of intervention are thus required to explore the potential longitudinal relationships between fNIRS complexity and the endurance performance. Meanwhile, the pre-processing of fNIRS data is important for obtaining valid and reliable hemodynamic signals as the fNIRS recording, especially when performing tasks (e.g., cycling), often consists of noise due to the interference from the task (e.g., the body motion) and the fluctuations that are not related to the hemodynamics (e.g., global physiological noise from the superficial tissues of the scalp). Therefore, advanced pre-processing techniques are highly-demanded in this field to help improve the validity of the data processing and interpretate and confirm the observations. Nevertheless, this study provides novel evidence of the influences of endurance exercise on the multiscale regulation of the PFC oxygenation, which can be captured by fNIRS complexity.

## 5 Conclusion

This study suggests that intaking the hydrogen gas before exercise can induce a significantly greater increase of fNIRS complexity, revealing the benefits of hydrogen gas for the facilitation of more complex regulation of PFC hemodynamics, which is critical to a better performance of the endurance task. These exciting findings suggest that the fNIRS complexity may serve as a marker of the integrity of multiple interacting physiologic mechanisms that regulate the PFC hemodynamics and is sensitive to both significant and subtle changes in this important function as induced by endurance exercise and interventions.

## Data Availability

All data in support of the findings of this paper are available within the article.
